# Change of urban park usage as a response to the COVID-19 global pandemic

**DOI:** 10.1038/s41598-023-46745-1

**Published:** 2023-11-07

**Authors:** Haokai Zhao, Brian J. Mailloux, Elizabeth M. Cook, Patricia J. Culligan

**Affiliations:** 1 Department of Civil Engineering and Engineering Mechanics, Columbia University, New York, NY 10027 USA; 2 Department of Environmental Science, Barnard College, New York, NY 10027 USA; 3 College of Engineering, Univerisity of Notre Dame, Notre Dame, IN 46556 USA

**Keywords:** Civil engineering, Environmental impact, Psychology and behaviour, Socioeconomic scenarios, Sustainability

## Abstract

Urban parks became critical for maintaining the well-being of urban residents during the COVID-19 global pandemic. To examine the impact of COVID-19 on urban park usage, we selected New York City (NYC) and used SafeGraph mobility data, which was collected from a large sample of mobile phone users, to assess the change in park visits and travel distance to a park based on 1) park type, 2) the income level of the visitor census block group (visitor CBG) and 3) that of the park census block group (park CBG). All analyses were adjusted for the impact of temperature on park visitation, and we focused primarily on visits made by NYC residents. Overall, for the eight most popular park types in NYC, visits dropped by 49.2% from 2019 to 2020. The peak reduction in visits occurred in April 2020. Visits to all park types, excluding Nature Areas, decreased from March to December 2020 as compared to 2019. Parks located in higher-income CBGs tended to have lower reductions in visits, with this pattern being primarily driven by large parks, including Flagship Parks, Community Parks and Nature Areas. All types of parks saw significant decreases in distance traveled to visit them, with the exception of the Jointly Operated Playground, Playground, and Nature Area park types. Visitors originating from lower-income CBGs traveled shorter distances to parks and had less reduction in travel distances compared to those from higher-income CBGs. Furthermore, both before and during the pandemic, people tended to travel a greater distance to parks located in high-income CBGs compared to those in low-income CBGs. Finally, multiple types of parks proved crucial destinations for NYC residents during the pandemic. This included Nature Areas to which the visits remained stable, along with Recreation Field/Courts which had relatively small decreases in visits, especially for lower-income communities. Results from this study can support future park planning by shedding light on the different uses of certain park types before and during a global crisis, when access to these facilities can help alleviate the human well-being consequences of “lockdown” policies.

## Introduction

Urban parks provide a variety of ecosystem services^1–3^, as well as physical and mental health benefits to urban residents^4–6^. The COVID-19 global pandemic drastically altered people’s mobility patterns^7–9^, especially during the first several months when restrictions were implemented by governments to combat the spread of the disease. Such restrictions included stay-at-home orders, the closure of non-essential businesses, the cancelation of public events and in-person schooling, social distancing, and travel restrictions, etc.^10–12^. With the many challenges imposed by the pandemic, which included dramatically reduced recreational opportunities as well as widespread concerns about personal and public health, urban parks, which were one of the few places that urban dwellers were allowed to visit outside their homes, became important destinations.

In order to understand the role of parks during the COVID-19 pandemic, a variety of data and methods have been used by researchers to conduct relevant studies: including carrying out field surveys^10,13,14^, recruiting civic scientists to make observations^15,16^, collecting geotagged data from social media^17–20^, and acquiring data from recreational tracking apps^21^. Decreased visits to urban greenspaces in central London was reported^20^, which could be attributed with working from home restrictions; similarly, in a study conducted in multiple cities across North Carolina, 56% of survey respondents indicated that they had ceased or reduced their use of parks, with geo-tracked park visits dropping by 15%^14^. Several studies reported increased visits to urban greenspaces and nature parks away from city centers^10,20,21^, while one study conducted in four Asian cities indicated people’s preference for large nature parks close to city centers^18^. The heightened appreciation for nature and the raised awareness of its importance have been highlighted by many studies^10,13,19^. Nonetheless, the parks studied were usually only loosely categorized, such as urban parks and nature parks; and one major concern about methods used in these studies, is that the data might not represent the general population very well because of the limited sample sizes.

SafeGraph, a location-based product company founded in 2016, has been collecting and compiling anonymous GPS data from mobile phone apps, and aggregating these data to provide information on people’s mobility patterns^22^. SafeGraph’s primary product is a *places* dataset of millions of points of interests (POIs) in the United States and Canada, providing details like business name, address, category, and geographic coordinates for each POI. They also provide a *patterns* dataset with anonymized, aggregated foot traffic data for those POIs. Because of the pervasiveness of smart phones in modern life, such data provides an opportunity for analyzing the mobility patterns of the general public. Several studies have employed SafeGraph data to investigate patterns of human mobility and spatial interaction, as well as their relationships with the spread of COVID-19^23–27^.

The goal of this study was to use the SafeGraph dataset to understand how park usage changed during the early phases of the COVID-19 pandemic, including by park location, detailed park type, and the socio-economic level of the park visitors and of the neighborhood of the park. Given the extensive size of the mobility dataset, we selected New York City (NYC) as our study area. NYC is representative of many dense, urban environments and was one of the major cities affected by the first waves of the pandemic. We examined changes in the number of visits to parks, the number of individual visitors to parks and the change in travel distance to parks. Our focus was primarily on park usage by NYC residents, rather than visitors to the city. Understanding how park usage changed during the pandemic is considered critical to the planning and management of urban public spaces in post-COVID cites, and in an era where future pandemics, and other crises that might lead to public “lockdowns”, cannot be ruled out^28^.

## Results

### The types of parks chosen for analysis

The total number of park visits determined from SafeGraph points of interest (POIs) located in NYC parks was 20,913,290 in 2019, but only 10,279,798 in 2020, representing a decrease of 49.2 percent.

**Figure 1:**
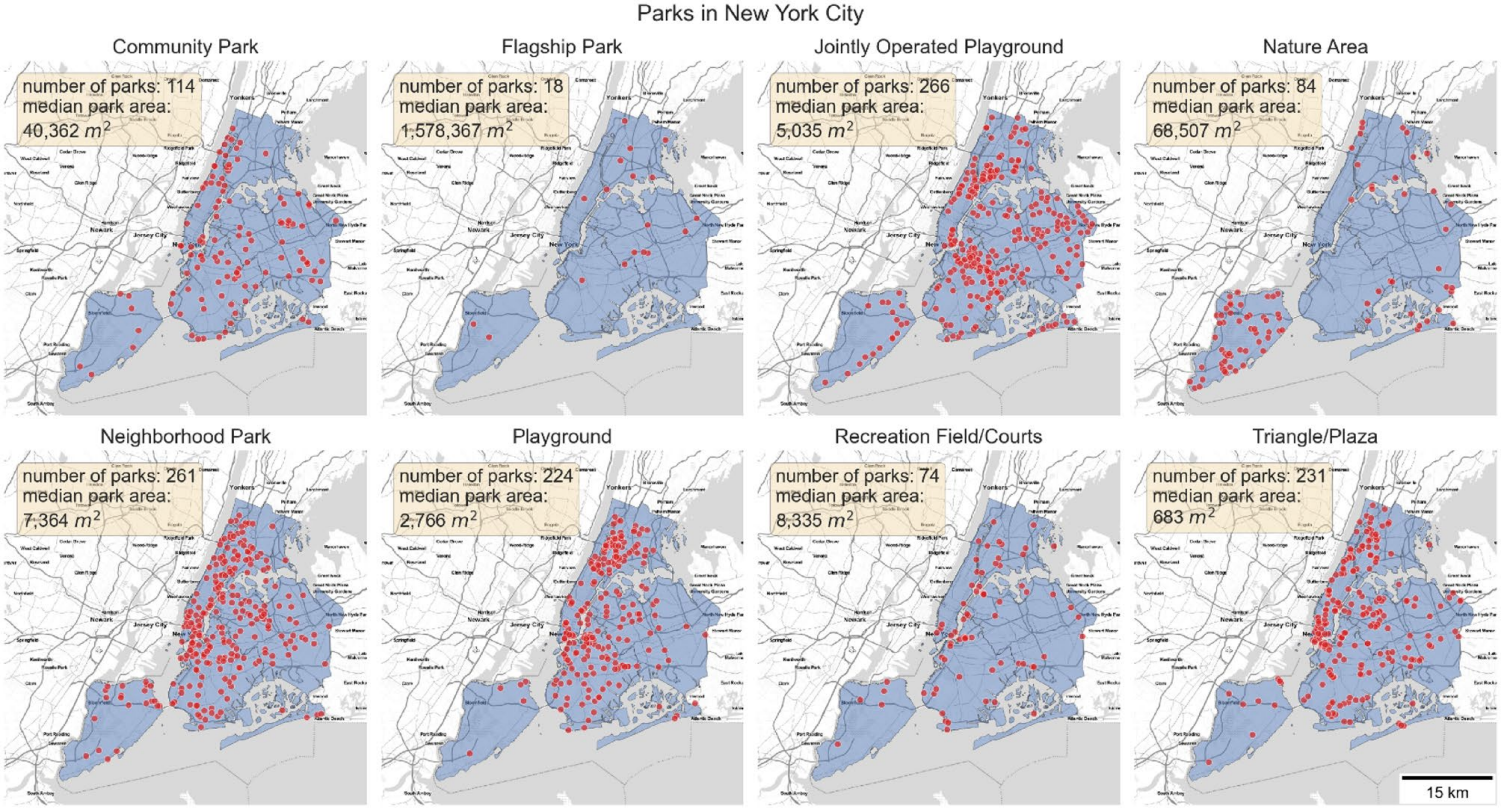
Locations of parks in New York City. Each point represents an individual park. The number of parks and the median park area are shown for each park type.

There are 18 types of parks listed in the NYC Open Space Parks Data^29^. However, the top eight types of parks accounted for 91.35% of total park visits in 2019 and 92.17% in 2020, respectively, and are thus the focus of this study (Fig. 1). These parks are classified by the NYC Department of Parks and Recreation^29^ as (1) Community Park, (2) Flagship Park, (3) Jointly Operated Playground, (4) Nature Area, (5) Neighborhood Park, (6) Playground, (7) Recreation Field/Courts, and (8) Triangle/Plaza. The detailed classification standard can be found in Table S-7.

We calculated four metrics associated with the number of park visits and the number of park visitors, namely (1) all visits: the total number of visits from all visitors; From the SafeGraph documentation, the duration of a visit must last at least 4 min, and there could be multiple visits from a single visitor during the time period when the data were collected; (2) all visitors: the total number of unique visitors, regardless of their origin; (3) US visitors: the total number of unique visitors whose home locations are within the US; (4) NYC local visitors: the total number of unique visitors whose home locations are within NYC. Since temperature has been reported as a vital factor influencing park visitation^30,31^, we corrected the data for the effects of temperature, as described in the “Data and methods” section. The total numbers of these four types of visit/visitor counts by park type, and after the temperature correction, are summarized in Table S-1.

### Park visits and visitors change rate

#### Park visits and visitors change rate by borough

**Figure 2:**
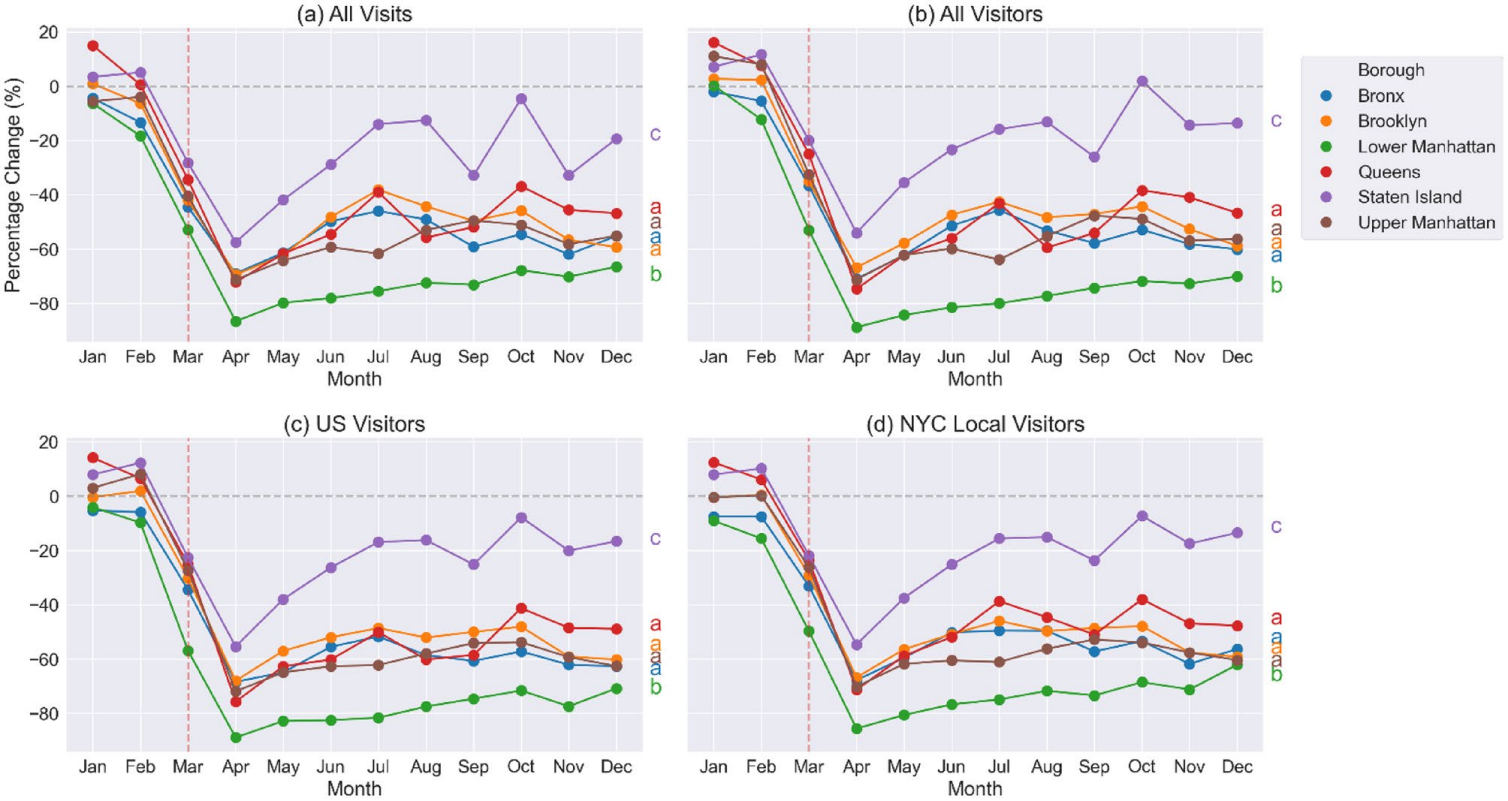
Park visits and visitors change rate by borough, which is calculated as the percent change of total monthly park visits/visitors in 2020 compared to 2019. The letters on the right of each figure are the Tukey HSD multi-group comparison results, the same letters indicate the boroughs belong to the same group.

We examined the park visits and visitors change rate in each NYC borough by computing the total number of park visits or visitors in a month in that borough, then calculating the percentage change in 2020 visits (or visitors) compared to 2019 (i.e., (the visits in 2020—the visits in 2019)/the visits in 2019). Manhattan was divided into lower Manhattan and upper Manhattan using 86th street as a divide. Results for total visits, total visitors, US visitors, and NYC visitors are shown in Fig. 2.

Starting from March 2020, the parks in all boroughs experienced decreased total visits (Fig. 2a). April 2020 was the month with the greatest percentage decrease in visits compared to 2019, then visits slowly increased as the months progressed. Lower Manhattan had the greatest decrease in park visits from March to December (overall − 61.1%, with a maximum of − 86.6% in April 2020), while Staten Island experienced the smallest decrease (overall − 20.3%, with a maximum of − 57.5% in April 2020). All other boroughs experienced similar changes in visits, and shared a similar trend through time. All visitors (Fig. 2b), U.S. visitors (Fig. 2b), and NYC visitors (Fig. 2d) had the same pattern with the greatest decrease in April, followed by a slow rebound with progressing time. Again, lower Manhattan had the largest decrease in unique visitors while Staten Island the smallest (Fig. 2b–d).

#### Park visits and visitors change rate by park type

**Figure 3:**
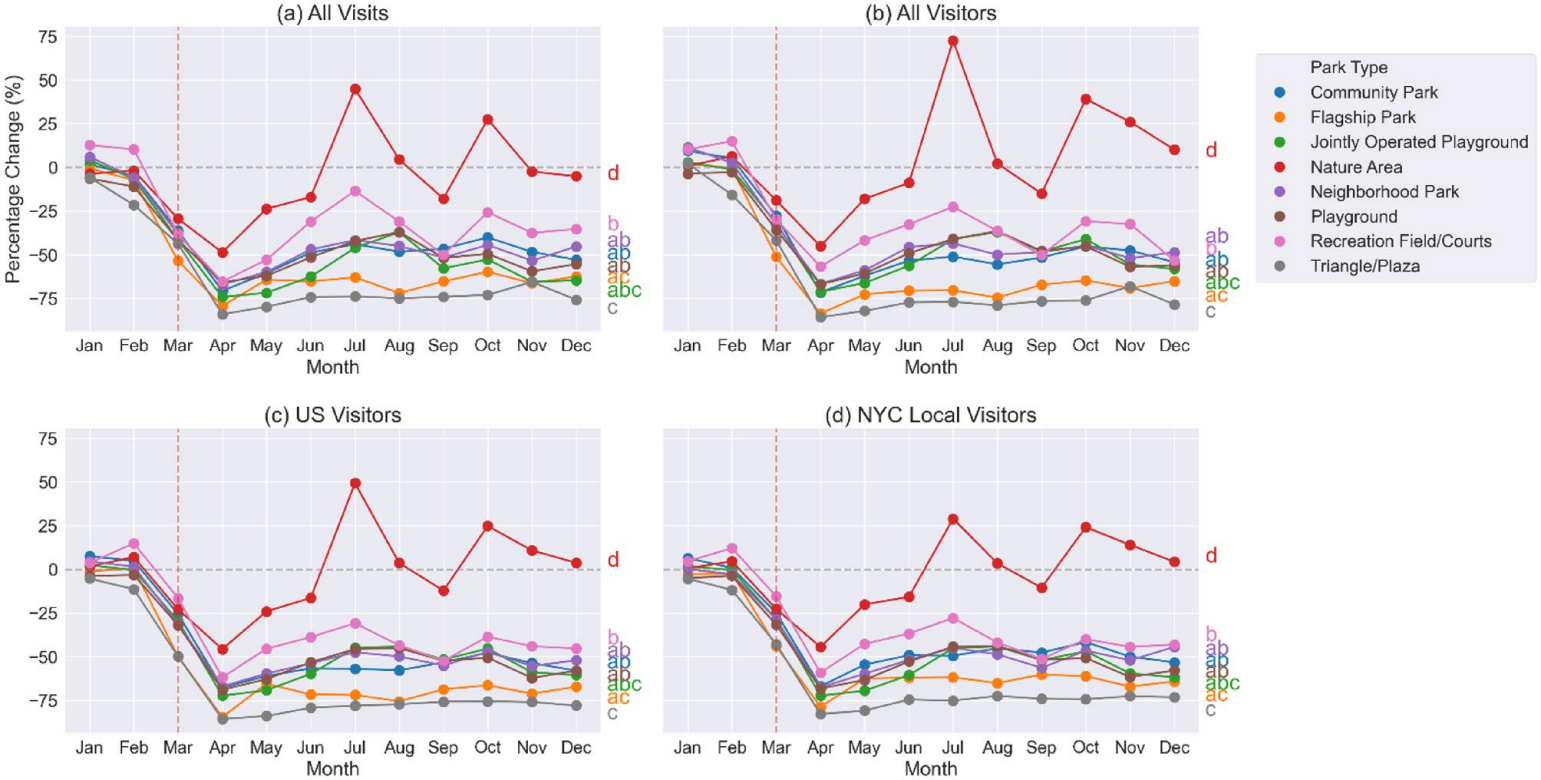
Park visits and visitors change rate by park type, which is calculated as the percent change of total monthly park visits/visitors in 2020 compared to 2019. The letters on the right of each figure are the Tukey HSD multi-group comparison results, any common letter shared by two park types indicates that the two park types were found to belong to the same group.

We examined the park visits and visitors change rate across the eight selected park types, by computing the total number of visits or unique visitors in a month to each park type and then calculating the percentage change of visits/visitors in 2020 compared to 2019 (Fig. 3). There was a decrease in all types of visits and visitors to all eight park types across the city when comparing 2019 to 2020 for the months of March to June (Fig. 3a–d). For NYC local visitors, Triangle/Plazas (overall − 62.9%, with a maximum of − 82.9% in April 2020) and Flagship Parks (overall − 57.0%, with a maximum of − 78.7% in April 2020) had the largest decrease, followed by Jointly Operated Playground, Playground, Community Park, Neighborhood Park, then Recreation Field/Courts. Nature Areas had the smallest decrease in the number of NYC local visitors (overall − 3.6%, with a maximum of − 44.5% in April 2020) with some months even showing an increase. Beginning in June, the number of NYC local visitors to Nature Areas returned to about the same level as 2019 and even increased in some months (with a maximum increase of 29.0% in July 2020) (Fig. 3d). The other three types of visits/visitors shared similar trends as NYC local visitors (Fig. 3a–c).

In order to better understand the needs and park usage of local urban residents, we focused our remaining analyses on data for NYC residents (also subsequently referred to as NYC local visitors) only. We also defined the visitor census block group (visitor CBG) as the home census block group where a visitor lived; and defined the park census block group (park CBG) as the census block group that a park was in, or was the closest to.

#### Park visitors change rate by both park type and by income level of park CBGs

**Figure 4:**
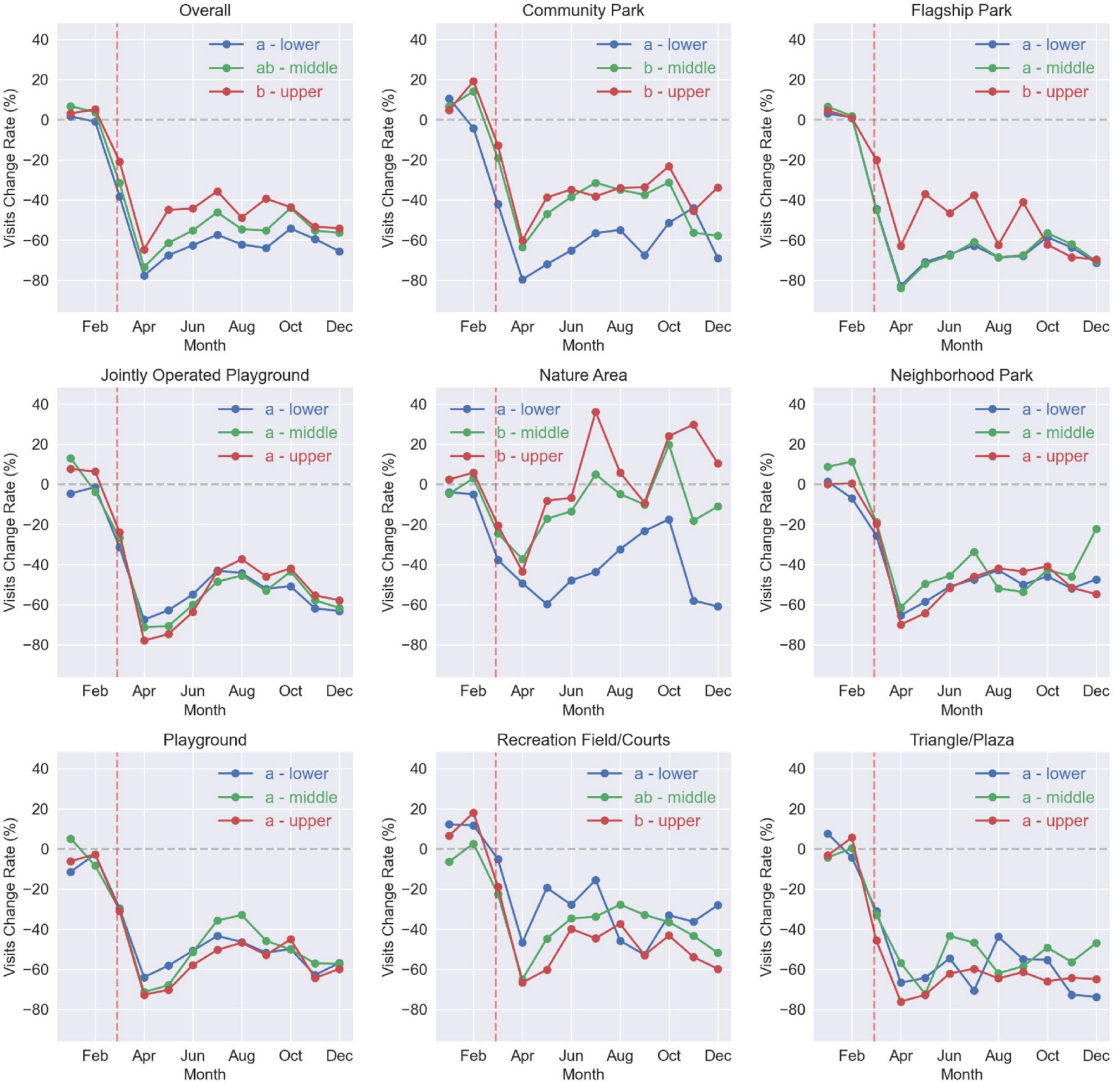
NYC park visitors change rate by park type and by income level of park CBGs. The letters before the income groups are the Tukey HSD multi-group comparison results, any common letter shared by two income level groups indicates that the two groups were found to belong to the same group.

The CBGs (neighborhoods) that surround parks were divided into three income groups: lower, middle and upper, based on per capita income. The results for park visits change rate between 2019 and 2020 for each of the analyzed park types are provided in Fig. 4.

All eight park types saw decreased NYC local visitors regardless of the park CBG income level (Fig. 4). Overall, parks in lower-income neighborhoods experienced statistically greater decreases in NYC local visitors than those in upper-income neighborhoods. No trend in visits change rate with income level was observed for Jointly Operated Playground, Neighborhood Park, Playground, and Triangle/Plaza. Community Parks and Nature Areas showed greater reductions in NYC local visitors in lower-income neighborhoods but showed no difference between middle- and upper-income neighborhoods. Flagship Parks showed greater reductions in NYC local visitors in lower- and middle-income neighborhoods. The outlier to the overall trend is Recreational Field/Courts, which showed greater reductions in NYC local visitors in upper-income neighborhoods than in lower-income neighborhoods.

### Travel distance to the parks

The travel distance of visitors was used to examine how the travel behavior of NYC residents to parks changed during the early stages of the COVID-19 pandemic. In this section, the mean travel distances were computed for the time period from March to December in 2019 and 2020, as the major outbreak of the pandemic and the associated travel restrictions began in March 2020.

#### Change in travel distance by park type

**Figure 5:**
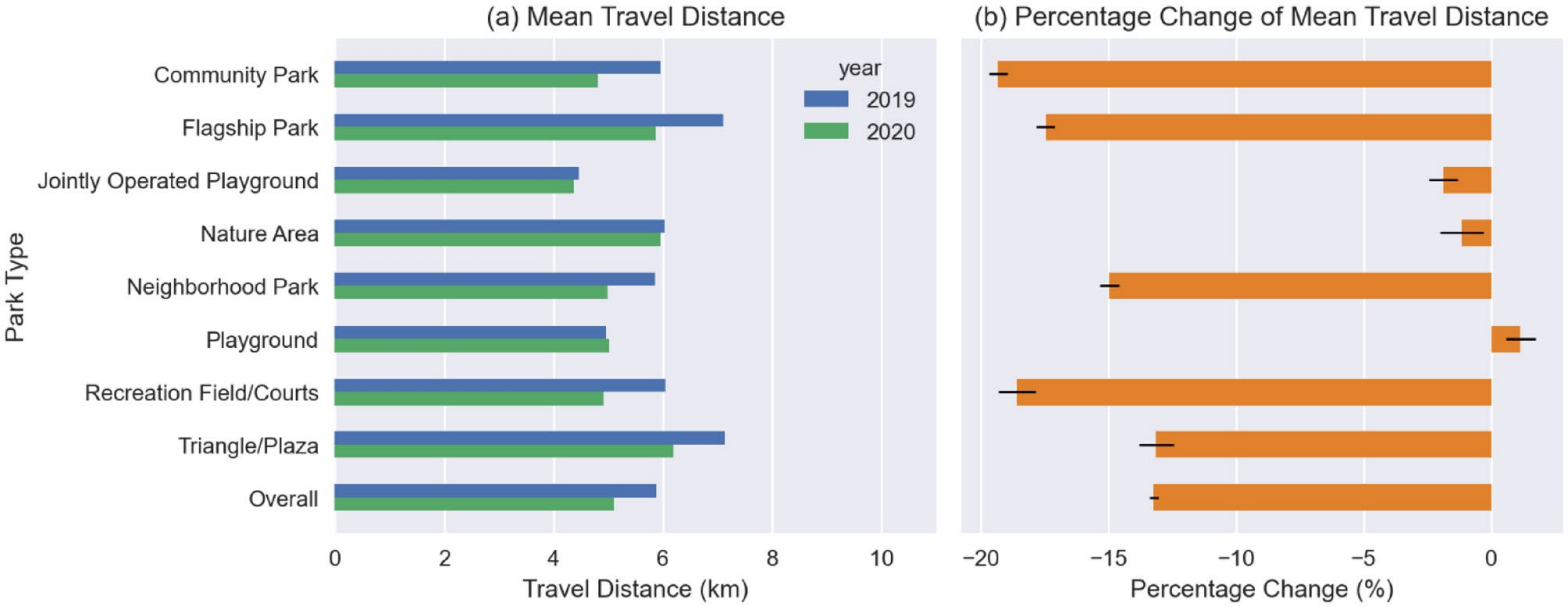
(**a**) Mean travel distance by park type. (**b**) Percentage change of mean travel distance by park type, with 95% CI error bars.

Overall, the mean travel distance of NYC residents to all parks reduced from 5.9 km in 2019 to 5.1 km in 2020 over March to December, representing a change of − 13.2% (95% CI − 13.4%, − 13.1%). In 2020, there was a significant decrease in travel distance compared to 2019 for all study park types except for the Jointly Operated Playground, Playground, and Nature Area park types (Fig. 5).

Before the pandemic, the mean travel distances to the Triangle/Plaza and Flagship Park types were the longest, both averaging 7.1 km—over March to December in 2019; while the travel distances to the Playground and Jointly Operated Playground were the shortest, averaging 5.0 km and 4.5 km, respectively. The Nature Area, Jointly Operated Playground and Playground park types experienced a smaller decrease than average or even a slight increase, which were − 1.2% (95% CI − 2.0%, − 0.3%), − 1.9% (95% CI − 2.5%, − 1.3%) and 1.1% (95% CI 0.6%, 1.7%), respectively. All other types of parks experienced a greater reduction in travel distance (Table S-4).

#### Change in travel distance by both park type and by income level of visitor CBGs

**Figure 6:**
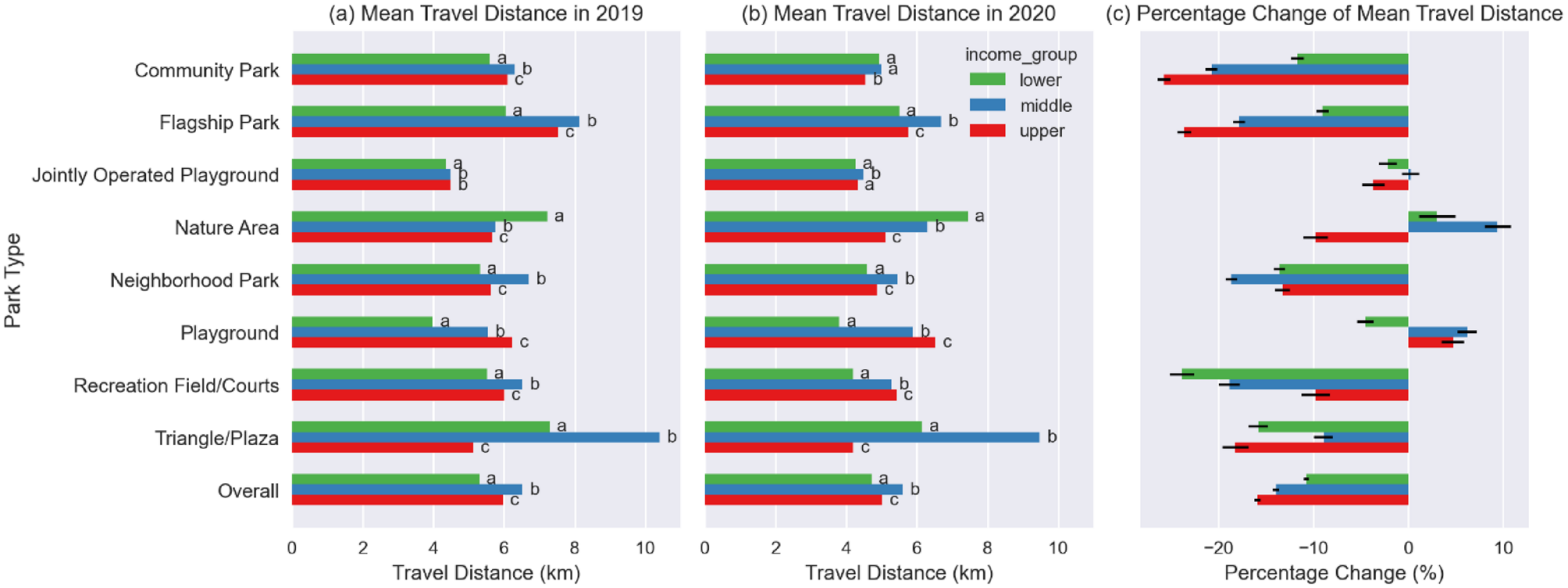
(**a**,**b**) Mean travel distance by park type and by income level of visitor CBGs; The letters to the right of the mean travel distances are the Tukey HSD multi-group comparison results between income groups for each park type, any common letter shared by two income groups indicates that the two groups were found to belong to the same group. (**c**) Percentage change of mean travel distance by park type and by income level of visitor CBGs, with 95% CI error bars.

The overall mean travel distances of NYC residents from lower-, middle- and upper-income level CBGs were 5.3 km, 6.5 km, and 6.0 km, respectively, from March to December in 2019; and were 4.7 km, 5.6 km, and 5.0 km, respectively, in the same period in 2020 (Fig. 6a,b). In general, people from lower-income CBGs traveled a statistically shorter distance to parks than those from middle-income and upper-income CBGs in both 2019 and 2020. This pattern was common across all types of parks, except for Nature Areas and Triangle/Plazas, to which visitors from upper-income CBGs traveled the shortest distance.

Overall, visitors from higher income CBGs had the greatest reduction in travel distance (Fig. 6c). The percentage change of travel distance for visitors from lower-income, middle-income and upper-income CBGs are − 10.7% (95% CI − 11.0%, − 10.4%), − 13.9% (95% CI − 14.2%, − 13.7%), and − 15.8% (95% CI − 16.2%, − 15.5%), respectively (Table S-5).

The specific changes varied by park type. For Community Park, Flagship Park, Jointly Operated Playground, Nature Area and Triangle/Plaza park types, the visitors from upper income level CBGs experienced the greatest percentage reduction in travel distance. While for Recreation Field/Courts, the visitors from upper income level CBGs had the smallest percentage reduction in travel distance.

#### Change in travel distance by both park type and by income level of park CBGs

**Figure 7:**
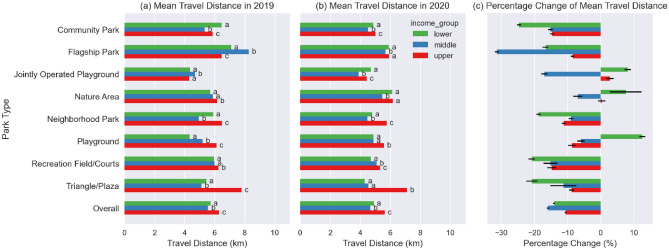
(**a**,**b**) Mean travel distance by park type and by income level of park CBGs; The letters to the right of the mean travel distances are the Tukey HSD multi-group comparison results between income groups for each park type, any common letter shared by two income groups indicates that the two groups were found to belong to the same group. (**c**) Percentage change of mean travel distance by park type and by income level of park CBGs, with 95% CI error bars.

The mean travel distances to parks located in lower-, middle- and upper-income level CBGs were 5.7 km, 5.6 km, and 6.3 km, respectively, from March to December in 2019, and were 4.9 km, 4.7 km, and 5.6 km, respectively, during the pandemic from March to December in 2020 (Fig. 7a,b). In general, people tended to travel a statistically longer distance to parks in upper-income CBGs than to parks in middle-income and lower-income CBGs in both 2019 and 2020, with the exception of Community Park and Flagship Park in 2019.

The travel distance to parks located in upper-income level CBGs had the smallest percentage decrease (Fig. 7c), which was − 10.6% (95% CI − 10.9%, − 10.3%), while it was − 16.0% (95% CI − 16.3%, − 15.7%) for parks located in middle-income CBGs, and was − 14.1% (95% CI − 14.4%, − 13.8%) for parks located in lower-income CBGs (Table S-6).

Examining by park type, for Community Park, Flagship Park, Recreational Field/Courts and Triangle/Plaza park types, the parks located in upper-income level CBGs experienced the smallest percentage reduction in travel distance. While for Nature Area, Jointly Operated Playground and Playground park types, the parks located in lower income level CBGs had an increase in travel distance.

## Discussion

### Change in the number of park visits and visitors

The data show a sharp decrease in park visits and visitors following the start of New York City related COVID-19 pandemic restrictions in March 2020, which for most parks lasted throughout the year. Across the boroughs, we observed that Lower Manhattan experienced the largest decrease in park visits and visitors. As Lower Manhattan is NYC’s central area for business, culture, and government, it is to be expected that visitation to this area decreased substantially after the pandemic restrictions were imposed. In contrast, Staten Island, which is more suburban with fewer tourist attractions, experienced the smallest reduction in park visits and visitors. By park type, Triangle/Plaza and Flagship Park saw the biggest decrease in visits and visitors among all park types. Triangles/Plazas are smaller park areas and are mostly located in densely populated business areas, the significant decrease in visits could be due to people not traveling to office buildings for work and therefore not using those Triangles/Plazas. Flagship Parks are destination attractions, even for NYC residents, and thus likely saw fewer visitations because people reduced destination-like leisure activities. Nature Areas had the smallest reduction in park visits and visitors, and even saw some increases above pre-pandemic level during summer months. Nature Areas are typically located on the outskirts of NYC or Staten Island (Fig. 1), where concerns about crowding are reduced^32^. The many services and benefits Nature Areas provide, from exercising, nature viewing, and birding, have also been shown to promote stress relief and mental health support during the pandemic^10,33,34^. From a policy perspective, certain types of parks in NYC were ordered closed from April through June, including Playgrounds (Fig. S-3) and Jointly Operated Playgrounds (jointly operated with the NYC Department of Education, Fig. S-4). The biggest decrease in park visits did occur in April 2020, however, the data showed a gradual rebound of visits to these parks while the order was in effect until summer months when temperatures may be too high for outdoor activities (Fig. 3), indicating that compliance with the lockdown policy may have declined overtime. In addition, as per observed trends for different types of park visits and visitors are similar, there is no indication that NYC residents behaved differently in terms of park visitation than non-NYC residents.

### Change in park visits by NYC residents based on income level of park CBGs

The decision to classify parks by income level was based on the fact that, neighborhood income was found to be an influential factor that impacts park usage in previous studies^35–37^. Furthermore, while many parks are designed to serve their immediate neighborhood, certain types with bigger sizes, such as Flagship Park, Community Park and Nature Area, could attract people from their larger service areas that may span the entire metropolitan region (Table S-7). This means that visitors may travel to parks in neighborhoods with different income levels than their own.

We observed that, parks located in higher-income neighborhoods experienced a smaller decrease in visits by NYC residents. This pattern was mainly driven by Flagship Park, Community Park and Nature Area, which are the three types of parks that usually have much larger areas (Fig. 1, Fig. S-2 and Table S-7); they saw greater reduction in visits in lower-income neighborhoods. Since park size was usually positively related with park use^38,39^, and wealthier neighborhoods are usually considered safer^40–43^; the combination of larger park areas, where social distancing is easier, located in wealthier neighborhoods likely explains this trend.

Recreation Field/Courts, which consist solely of hard surface and turf sports areas, showed a different trend, in that parks located in lower-income neighborhoods experienced a smaller decrease in visits by NYC residents. In many low-income communities, community located recreation facilities are often the only place for children to be physically active^35^, which might explain greater usage of these facilities in these neighborhoods. For the rest of the types of parks, there was no significant difference in park visits change rate by NYC residents between income levels.

### Change in travel distance to parks for NYC residents

Through our analysis, we observed that mean travel distance to urban parks by NYC residents decreased from 5.9 km before the pandemic to 5.1 km during the pandemic. More specifically, there was a significant decrease in travel distance for all park types except Nature Area, Jointly Operated Playground, and Playground where travel distances remained similar to pre-pandemic levels. As previously discussed, Nature Area served as a safe haven for urban residents during the pandemic, with visits remaining relatively stable and even increasing in some months. People were willing to continue to travel to visit these areas despite the fact that they were frequently located on the fringes of the city^20,21,44^. Jointly Operated Playground and Playground are scattered throughout the city's residential zones. Prior to the pandemic, travel distances to these two types of parks were the shortest among all types, implying that their primary role was to serve local residents. During the pandemic, visitors to them are still likely local residents, resulting in a small change in travel distance.

Visitors from lower-income CBGs tended to travel a statistically shorter distance to parks than those from middle-income and upper-income CBGs. This could be explained by the fact that, people with higher incomes were willing to spend money on transportation and, as a result, were more able to travel to parks located a further distance away. Travel distances for visitors from upper-income CBGs decreased the most among the three income groups; this could be attributed to the fact that people with higher socio-economic status were more likely to have the ability to work from home^45–47^.

When considering the income level of park CBGs, in general, people tended to travel a statistically longer distance to the parks located in upper-income level CBGs, and the travel distance to those parks decreased the least in percentage compared to parks in middle-income and lower-income CBGs. As high-income neighborhoods are usually perceived as a safer environment^40–43^, this might explain people’s willingness to travel longer distances to the parks located there, even during the pandemic, resulting in a smaller reduction in travel distance.

First observations, the travel distances by NYC residents appear to be longer than expected, however, from other studies, we found comparable travel distance ranges. For example, one study conducted in Sapporo, Japan investigated the travel distances to urban parks and nature trails; they reported a mean travel distance to urban parks of 6.8–11.0 km before the pandemic and 4.9–10.6 km after the pandemic^48^. Another study conducted in Wuhan, China found that the threshold travel distance (TTD, i.e., the third quartile travel distance for all visitors) to urban parks reduced from 4.2 km in pre-pandemic time to 3.0–3.9 km during different stages of the pandemic; TTD varied by travel modes, it ranged from about 1.1 km for walking to about 15 km for visitors taking buses and subways^49^. The mean travel distance reported in our study reflected the general situation for all visitors with mixed travel modes, future studies can further disaggregate travel distances by travel modes when such information becomes available.

### The importance of nature areas and recreation field/courts

Throughout the analysis from all these aspects, we would like to highlight Nature Areas, as they were the only type that saw a slight decrease or even some increases in visits and, furthermore, the travel distance to them also remained relatively stable. Perceived as a safe haven, these results demonstrated Nature Area’s critical role of serving urban residents in a time of crisis, and they should be well planned and maintained in the future urban developments. We would also like to highlight Recreation Field/Courts, particularly their significance in serving lower-income communities. They experienced a smaller decrease in visits than most types of parks except for Nature Areas, and they were the only park type that saw a greater decrease in visits in upper-income CBGs and less decrease in lower-income CBGs. Travel distances to them have also fallen significantly, with the biggest decrease coming from people living in lower-income CBGs. These findings suggested that Recreation Field/Courts probably became the key destinations for local people during the pandemic, especially for those living in lower-income neighborhoods.

### Limitations and avenues for future research

Although we adjusted the park visits for temperature, which was a major factor in influencing park usage, other potential sources of bias in the data could exist. The number of devices in SafeGraph’s panel, for example, may change over time, introducing biases in visit counts; this issue could be especially prominent in cross-region studies^50,51^. To overcome this bias, SafeGraph has recommended several methods for normalization^52^. Since the supplementary datasets for normalization were not available at the time when we acquired the data for the analyses presented in this paper, we used the normalization by total visitors method to assess this potential bias in a secondary analysis. Overall, the normalized visits data were highly correlated with the raw visits data (Pearson’s R = 0.980, Table S-8). For individual park types, all park types showed a high correlation coefficient of over 0.9, except for Nature Area (Pearson’s R = 0.763). From further inspection, it appears that the raw visits data may have overestimated the visits to Nature Areas in 2019 or have underestimated the visits in 2020 (Fig. S-5), implying the percentage increase in visits to Nature Area in 2020 could be even greater than reported here. While this does not violate our general conclusions, future research should consider this potential bias and make adjustments accordingly.

Our data showed that park neighborhood income level was a significant factor correlated with the park usage during the pandemic, however, it could be indicative of multiple factors that influence the choice to visit a park. Previous studies have found that parks in lower-income neighborhoods can be associated with poorer facilities, fewer services and less maintenance^35,37^. Independent of neighborhood income level, park features such as sports facilities and water scenes, were found to be positively associated with park use^20,53^; factors describing the surrounding environments of parks, such as population density, road density, distance to city center and accessibility via public transportation can also impact park usage^38,54^. Future studies should investigate the underlying factors that are indicated by neighborhood income and influence park usage, therefore providing more detailed insights that could be used to improve park service.

Our findings indicate that there has been a fundamental change in park visitation habits of urban residents, and the change varies by park type and socio-economic status. Future studies should explore more in detail how other factors could potentially affect people’s park visitation, such as age, gender, ethnicity and means of transportation, etc. Furthermore, the identified specific park types, such as Nature Area, for its importance to general urban residents; and Recreational Field/Court, especially for its importance to lower-income communities, should be well planned and managed to make urban parks a more resilient infrastructure system in the face of future crisis.

## Data and methods

We obtained the mobility data from SafeGraph^55^ for the time period of January 2018 to December 2020. SafeGraph aggregates anonymized location data from numerous applications in order to provide insights about points of interest (POIs) that people visit, via the SafeGraph Community. To enhance privacy, SafeGraph excludes census block group (CBG) information if fewer than two devices visited an establishment in a month from a given census block group. As examined in our analysis that comparing 2020 data to 2018 yielded similar results to 2019, we chose to present comparisons of data from 2020 to 2019 for clarity.

SafeGraph’s *places* dataset contains a variety of information for each POI in its product suite, including the location name, address, latitude and longitude coordinates, category, brand, and additional details. To select those POIs of our interest, we obtained the NYC Open Space Parks Data^29^, which is a vector GIS dataset that provides information on park characteristics such as location, boundary, and park type. We then used the ArcGIS Pro software to spatially select those POIs within park boundaries. The NYC Open Space Parks Dataset was chosen because of its comprehensive coverage of the parks in the city, and the NYC Parks Department classified the parks into distinct types based on park features, service area and size, etc. (Table S-7).

SafeGraph designations don’t always match with NYC Parks Department designations. Some studies have focused on the SafeGraph designated POIs with type “Nature Parks and Other Similar Institutions”, as this approach unified the selection of parks across multiple cities^51^; however, they excluded some park types such as playgrounds and greenspaces that may be coded under other categories in the SafeGraph POIs dataset, such as “Museums, Historical Sites, and Similar Institutions” and “Other Amusement and Recreation Industries”, etc. Our approach included all these POIs that fall in the government-designated park boundaries and enabled comparisons between different types of parks in our studied urban area.

The *monthly patterns* dataset from SafeGraph contains visitors’ mobility information and is organized by POI. As mentioned in the types of parks chosen for analysis section, we calculated four types of visits: (1) all visits: to calculate the number of visits to each park, we summed *raw_visit_counts* to all the POIs within a park; (2) all visitors: *raw_visitor_counts* to all the POIs within a park were summed; (3) US visitors: the *visitor_home_cbgs* column provides the number of visitors to the POI from each US CBG based on the visitor's home location, they were summed to determine US visitors; (4) NYC local visitors: the visitors who are from a CBG within NYC were summed as the number of NYC local visitors. When deriving travel distances, we obtained the US Census Block Group (CBG) Boundaries GIS data^56^ from the US Census Bureau, then we calculated the distance between the centroid of a visitor’s home CBG and the destination POI.

Temperature has been reported as a vital factor influencing park visitation^30,31,57^. To adjust visits data by temperature, NYC daily and monthly climate data were downloaded from the NOAA climate database^58^ for the year of 2018 to 2020. The data included weather information from multiple weather stations throughout the city, which were then averaged to represent the citywide mean temperature. The relationship between monthly park visits and monthly mean temperature was confirmed using Pearson’s correlation coefficient. Overall, the number of park visits was found to be highly positively correlated with temperature (Pearson's R = 0.78, p < 0.001) for the time period from January 2018 to February 2020 (Table S-2), which was prior to the implementation of the COVID-19 policies restricting people's mobility in NYC. Except for jointly operated playgrounds, the relationship holds for all park types.

To adjust park visitation by temperature, we first conducted a paired t-test to compare NYC daily mean temperature in each month between 2019 and 2020. The temperature in 5 months was found to be significantly different in the two years (Table S-2), indicating the necessity for correction. Later, we built several models using the least squares method and the Gaussian Process method to model the relationship between park visits and temperature. The best one, a third-degree polynomial model with an R^2^ value of 0.72, was then used to adjust park visits data (Equation S-1 and S-2).

To investigate the impact of socioeconomic factors, the American Community Survey's annual average per capita income data^59^ at the CBG level for the year 2019 were obtained. We defined the visitor census block group (visitor CBG) as the visitor's home CBG, which was provided by the *visitor_home_cbgs* column in the *monthly patterns* dataset; and we defined the park census block group (park CBG) as the CBG in which the park was located or was the closest to, which was determined using ArcGIS Pro software by attributing the nearest CBG to each POI.

The income data were then combined with park visits data based on visitor CBGs and park CBGs, and the income groups were determined by the per capita income level of these CBGs, with the three terciles serving as the cutoff numbers for lower-, middle- and upper-income levels. For group comparisons, e.g. visits change rate between boroughs/park types, visits change rate between income groups for each park type, and travel distance between income groups, etc. Tukey’s HSD post-hoc test was used^60^.

### Supplementary Information

Supplementary Information.

## Data Availability

The smartphone mobility data that support the findings of this study are acquired from SafeGraph Inc. However, due to certain restrictions, these data, which were licensed for this specific study, are not openly accessible. Nevertheless, the authors can provide the data upon a reasonable request, subject to SafeGraph Inc.'s approval.

## Code availability

The other datasets and code that support this study's findings can be publicly accessed from the project's GitHub repository: https://github.com/hkzhao7/covid19-urban-park-usage.
