# Supplementary material for: Change of urban park usage as a response to the COVID-19 global pandemic
Source: Sci Rep. 2023 Nov 7;13:19324. doi: 10.1038/s41598-023-46745-1 (PMC10630328; doi:10.1038/s41598-023-46745-1)
Supplement: Supplementary file 1 — Supplementary Information. [file 41598_2023_46745_MOESM1_ESM.docx]

**Supplementary Information for:**

**Change of Urban Park Usage in New York City as a Response to the COVID-19 Global Pandemic**

Haokai Zhao^1^, Brian J. Mailloux^2^, Elizabeth M. Cook^2^, Patricia J. Culligan^3,*^

^1^Department of Civil Engineering and Engineering Mechanics, Columbia University, New York, NY, 10027, USA

^2^Department of Environmental Science, Barnard College, New York, NY, 10027, USA

^3^College of Engineering, Univerisity of Notre Dame, Notre Dame, IN, 46556, USA

^*^e-mail: pculliga@nd.edu

# Supplementary Information

### Summary of visit and visitor count by park type

Table S-1 Summary of visit and visitor count by park type

| Year | Park Type | NYC Local Visitor Count | US Visitor Count | All Visitor Count | All Visit Count | Local Visitor to US Visitor Fraction (%) | Local Visitor to All Visitor Fraction (%) |
| --- | --- | --- | --- | --- | --- | --- | --- |
| 2019 | Neighborhood Park | 1,302,203 | 1,716,881 | 2,513,057 | 4,704,165 | 75.9 | 51.8 |
|  | Community Park | 1,009,907 | 1,291,056 | 1,708,520 | 3,285,743 | 78.2 | 59.1 |
|  | Flagship Park | 842,038 | 1,336,529 | 1,732,384 | 3,277,968 | 63.0 | 48.6 |
|  | Jointly Operated Playground | 763,718 | 875,623 | 1,227,608 | 2,924,808 | 87.2 | 62.2 |
|  | Playground | 663,992 | 805,454 | 1,153,740 | 2,357,716 | 82.4 | 57.6 |
|  | Triangle/Plaza | 391,581 | 574,661 | 831,113 | 1,272,079 | 68.1 | 47.1 |
|  | Recreation Field/Courts | 218,660 | 260,763 | 352,750 | 733,724 | 83.9 | 62.0 |
|  | Nature Area | 147,566 | 179,116 | 228,543 | 550,484 | 82.4 | 64.6 |
| 2020 | Neighborhood Park | 593,175 | 727,438 | 1,071,939 | 2,118,963 | 81.5 | 55.3 |
|  | Community Park | 554,014 | 643,666 | 888,053 | 1,786,854 | 86.1 | 62.4 |
|  | Flagship Park | 381,410 | 500,057 | 673,322 | 1,380,409 | 76.3 | 56.7 |
|  | Jointly Operated Playground | 374,947 | 440,377 | 638,408 | 1,411,035 | 85.1 | 58.7 |
|  | Playground | 365,820 | 455,150 | 699,710 | 1,375,563 | 80.4 | 52.3 |
|  | Triangle/Plaza | 132,099 | 178,209 | 248,510 | 398,149 | 74.1 | 53.2 |
|  | Recreation Field/Courts | 134,515 | 156,466 | 215,054 | 469,251 | 86.0 | 62.6 |
|  | Nature Area | 148,904 | 181,178 | 241,645 | 535,313 | 82.2 | 61.6 |

### B. Temperature adjustment model for park visits

Table S-2 – Relationship between the number of local NYC park visitors and temperature

| **Park Type** | **Neighborhood Park** | **Community Park** | **Flagship Park** | **Jointly Operated Playground** | **Playground** | **Triangle/**  **Plaza** | **Nature Area** | **Recreation Field/Courts** | **Overall** |
| --- | --- | --- | --- | --- | --- | --- | --- | --- | --- |
| **Pearson’s R** | 0.66 | 0.85 | 0.92 | 0.11 | 0.65 | 0.68 | 0.52 | 0.62 | 0.78 |
| **p value** | <0.001 | <0.001 | <0.001 | 0.594 | <0.001 | <0.001 | <0.01 | <0.001 | <0.001 |

Note: the sample sizes are all 26 (the number of months starting from Jan 2018 to Feb 2020)

To determine if there was a significant difference in temperature patterns between 2020 and 2019, a paired t-test was conducted for each month. The results, presented in Table S-3, revealed that temperatures in January, February, March, April, and November showed significant differences between the two years.

Table S-3 Paired t-test results for monthly temperatures in 2020 and 2019

| **Month** | **1** | **2** | **3** | **4** | **5** | **6** | **7** | **8** | **9** | **10** | **11** | **12** |
| --- | --- | --- | --- | --- | --- | --- | --- | --- | --- | --- | --- | --- |
| **Sample Size** | 31 | 28 | 31 | 30 | 31 | 30 | 31 | 31 | 30 | 31 | 30 | 31 |
| **t stat** | 2.77 | 2.24 | 3.66 | -3.15 | -1.15 | 1.54 | 0.39 | 1.65 | -0.84 | -0.93 | 5.17 | 0.65 |
| **p value** | <0.01 | 0.03 | <0.001 | <0.01 | 0.26 | 0.13 | 0.70 | 0.11 | 0.41 | 0.36 | <0.001 | 0.52 |

Therefore, to remove the effect of temperature on park visits, several models were developed using the ordinary least squares (OLS) method and the Gaussian Process method, as shown in Figure S-1.


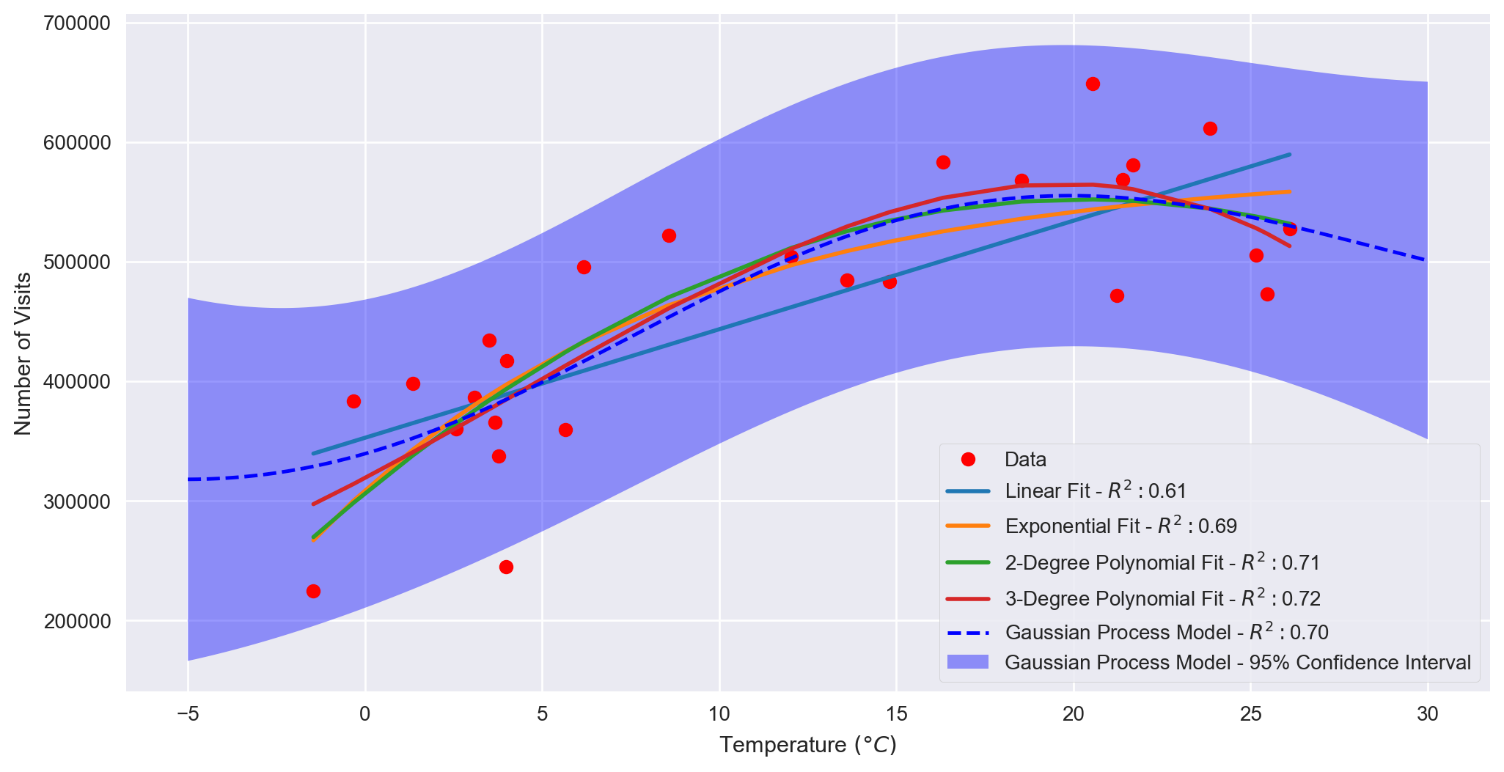


Figure S-1 Models of park visits vs temperature

The highest R^2^ value of 0.72 was achieved by a 3rd-degree polynomial model (Equation S-1), which was then employed to adjust the park visits data.

$f\left( t \right)=-24.4\cdot t^{3}-325.2\cdot t^{2}+15553.0\cdot t+318999.5$ (S-1)

Where *f(t)* is the total number of park visits in a month, and *t* is the average temperature in that month.

Equation S-2 was used to adjust the number of monthly visits to every single park:

$V_{base, adj}=V_{2019}\times1+[\frac{f\left( t_{2020} \right)-f\left( t_{2019} \right)}{f\left( t_{2020} \right)}]$ (S-2)

Where *V_2019_* is the number of visits in 2019, *t_2019_* and *t_2020_* are the monthly average temperatures in 2019 and 2020, respectively, and *V_base,adj_* is the assumed base number of visits after adjustment for temperature differences: this parameter can be interpreted as the number of visits to a park in a month of 2019 if the temperature in that month was the same as that in 2020.

The temperature adjustment model was developed for each of the four types of visits/visitors: 1) all visits, 2) all visitors, 3) US visitors, and 4) NYC local visitors, as described in the Data and Methods section. The model described in this section was developed using data for NYC local visitors; the modeling results by using the other three types of visits/visitors were all similar as the model provided here.

### C. The mean and change of travel distance by park type

Table S-4 Summary of the mean and change of travel distance by park type

| Park Type | Mean Travel Distance in 2019 (km) | Mean Travel Distance in 2020 (km) | Change of Travel Distance (km, 95% CI) | Change of Travel Distance (percentage, 95% CI) |
| --- | --- | --- | --- | --- |
| Overall | 5.9 | 5.1 | -0.8 (-0.8, -0.8) | -13.2 (-13.4, -13.1) |
| Triangle/Plaza | 7.1 | 6.2 | -0.9 (-1.0, -0.9) | -13.1 (-13.8, -12.4) |
| Flagship Park | 7.1 | 5.9 | -1.2 (-1.3, -1.2) | -17.5 (-17.8, -17.1) |
| Recreation Field/Courts | 6.0 | 4.9 | -1.1 (-1.2, -1.1) | -18.6 (-19.3, -17.8) |
| Nature Area | 6.0 | 6.0 | -0.1 (-0.1, 0.0) | -1.2 (-2.0, -0.3) |
| Community Park | 6.0 | 4.8 | -1.2 (-1.2, -1.1) | -19.3 (-19.7, -19.0) |
| Neighborhood Park | 5.9 | 5.0 | -0.9 (-0.9, -0.9) | -15.0 (-15.3, -14.6) |
| Playground | 5.0 | 5.0 | 0.1 (0.0, 0.1) | 1.1 (0.6, 1.7) |
| Jointly Operated Playground | 4.5 | 4.4 | -0.1 (-0.1, -0.1) | -1.9 (-2.5, -1.3) |

### D. The mean and change of travel distance by park type and by income level of visitor CBGs

Table S-5 Summary of the mean and change of travel distance by park type and by income level of visitor CBGs

| Park Type | Mean Travel Distance – 2019, 2020 (km) | | | Change of Travel Distance  and 95% CI (km) | | | Change of Travel Distance  and 95% CI (percentage) | | |
| --- | --- | --- | --- | --- | --- | --- | --- | --- | --- |
|  | lower | middle | upper | lower | middle | upper | lower | middle | upper |
| Overall | 5.3, 4.7 | 6.5, 5.6 | 6.0, 5.0 | -0.6  (-0.6, -0.6) | -0.9  (-0.9, -0.9) | -1.0  (-1.0, -0.9) | -10.7  (-11.0, -10.4) | -13.9  (-14.2, -13.7) | -15.8  (-16.2, -15.5) |
| Community Park | 5.6, 5.0 | 6.3, 5.0 | 6.1, 4.5 | -0.7  (-0.7, -0.6) | -1.3  (-1.3, -1.3) | -1.6  (-1.6, -1.5) | -11.7  (-12.4, -11.0) | -20.7  (-21.3, -20.1) | -25.7  (-26.4, -25.0) |
| Flagship Park | 6.1, 5.5 | 8.1, 6.7 | 7.5, 5.8 | -0.6  (-0.6, -0.5) | -1.5  (-1.5, -1.4) | -1.8  (-1.8, -1.7) | -9.0  (-9.7, -8.4) | -17.8  (-18.4, -17.2) | -23.6  (-24.3, -22.9) |
| Jointly Operated Playground | 4.4, 4.3 | 4.5, 4.5 | 4.5, 4.3 | -0.1  (-0.1, -0.1) | 0.0  (0.0, 0.1) | -0.2  (-0.2, -0.1) | -2.2  (-3.1, -1.2) | 0.2  (-0.7, 1.2) | -3.7  (-4.9, -2.5) |
| Nature Area | 7.2, 7.4 | 5.8, 6.3 | 5.7, 5.1 | 0.2  (0.1, 0.4) | 0.5  (0.5, 0.6) | -0.6  (-0.6, -0.5) | 3.0  (1.1, 5.0) | 9.4  (8.0, 10.8) | -9.8  (-11.1, -8.5) |
| Neighborhood Park | 5.3, 4.6 | 6.7, 5.5 | 5.6, 4.9 | -0.7  (-0.8, -0.7) | -1.3  (-1.3, -1.2) | -0.8  (-0.8, -0.7) | -13.6  (-14.2, -12.9) | -18.6  (-19.2, -18.0) | -13.3  (-14.0, -12.5) |
| Playground | 4.0, 3.8 | 5.6, 5.9 | 6.2, 6.5 | -0.2  (-0.2, -0.1) | 0.3  (0.3, 0.4) | 0.3  (0.2, 0.4) | -4.5  (-5.4, -3.6) | 6.2  (5.2, 7.2) | 4.7  (3.5, 5.9) |
| Recreation Field/Courts | 5.5, 4.2 | 6.5, 5.3 | 6.0, 5.4 | -1.3  (-1.4, -1.3) | -1.2  (-1.3, -1.2) | -0.6  (-0.7, -0.5) | -23.8  (-25.1, -22.6) | -18.9  (-20.0, -17.7) | -9.8  (-11.3, -8.3) |
| Triangle/Plaza | 7.3, 6.2 | 10.4, 9.5 | 5.1, 4.2 | -1.2  (-1.2, -1.1) | -0.9  (-1.0, -0.8) | -0.9  (-1.0, -0.9) | -15.8  (-16.8, -14.8) | -9.0  (-10.0, -8.0) | -18.2  (-19.6, -16.8) |

### E. The mean and change of travel distance by park type and by income level of park CBGs

Table S-6 Summary of the mean and change of travel distance by park type and by income level of park CBGs

| Park Type | Mean Travel Distance – 2019, 2020 (km) | | | Change of Travel Distance  and 95% CI (km) | | | Change of Travel Distance  and 95% CI (percentage) | | |
| --- | --- | --- | --- | --- | --- | --- | --- | --- | --- |
|  | lower | middle | upper | lower | middle | upper | lower | middle | upper |
| Overall | 5.7, 4.9 | 5.6, 4.7 | 6.3, 5.6 | -0.8  (-0.8, -0.8) | -0.9  (-0.9, -0.9) | -0.7  (-0.7, -0.7) | -14.1  (-14.4, -13.8) | -16.0  (-16.3, -15.7) | -10.6  (-10.9, -10.3) |
| Community Park | 6.5, 4.9 | 5.3, 4.5 | 5.9, 5.0 | -1.6  (-1.6, -1.6) | -0.8  (-0.9, -0.8) | -0.9  (-0.9, -0.8) | -24.6  (-25.2, -24.0) | -15.2  (-15.8, -14.5) | -14.7  (-15.3, -14.0) |
| Flagship Park | 7.1, 5.9 | 8.3, 5.7 | 6.5, 5.9 | -1.2  (-1.2, -1.1) | -2.6  (-2.6, -2.5) | -0.6  (-0.6, -0.5) | -16.6  (-17.5, -15.8) | -31.3  (-31.9, -30.7) | -8.5  (-9.0, -7.9) |
| Jointly Operated Playground | 4.4, 4.7 | 4.7, 3.9 | 4.3, 4.4 | 0.4  (0.3, 0.4) | -0.8  (-0.8, -0.8) | 0.1  (0.1, 0.2) | 8.2  (7.2, 9.2) | -17.0  (-17.9, -16.1) | 2.8  (1.7, 3.9) |
| Nature Area | 5.7, 6.1 | 5.9, 5.5 | 6.2, 6.2 | 0.4  (0.2, 0.7) | -0.4  (-0.5, -0.3) | 0.0  (-0.1, 0.1) | 7.6  (2.7, 12.4) | -6.9  (-8.3, -5.5) | 0.3  (-0.8, 1.4) |
| Neighborhood Park | 5.9, 4.8 | 4.9, 4.5 | 6.5, 5.8 | -1.1  (-1.1, -1.1) | -0.4  (-0.5, -0.4) | -0.7  (-0.8, -0.7) | -18.8  (-19.4, -18.1) | -9.0  (-9.7, -8.2) | -11.1  (-11.7, -10.5) |
| Playground | 4.4, 4.9 | 5.2, 4.9 | 6.1, 5.6 | 0.6  (0.5, 0.6) | -0.3  (-0.4, -0.3) | -0.5  (-0.6, -0.5) | 12.6  (11.7, 13.5) | -5.9  (-7.0, -4.8) | -8.7  (-9.8, -7.7) |
| Recreation Field/Courts | 6.0, 4.7 | 6.0, 5.1 | 6.2, 5.3 | -1.2  (-1.3, -1.2) | -0.9  (-1.0, -0.8) | -0.9  (-1.0, -0.8) | -20.8  (-21.8, -19.9) | -15.2  (-17.3, -13.1) | -14.8  (-16.1, -13.4) |
| Triangle/Plaza | 5.4, 4.3 | 5.1, 4.5 | 7.8, 7.1 | -1.1  (-1.2, -1.0) | -0.6  (-0.8, -0.4) | -0.7  (-0.7, -0.6) | -20.8  (-22.5, -19.0) | -11.2  (-15.2, -7.3) | -8.8  (-9.5, -8.0) |

### F. Auxiliary information about park characteristics


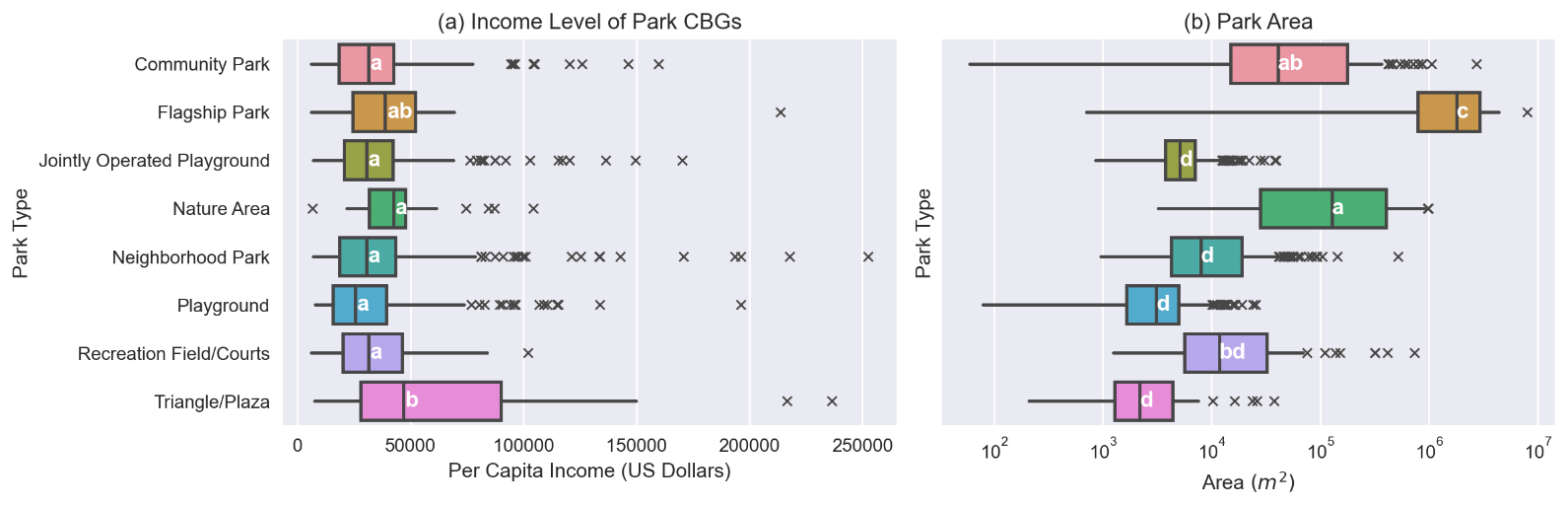


Figure S-2 (a) Income level of park CBGs by park type; (b) park area by park type. The letters inside the boxes are the Tukey HSD multi-group comparison results for each topic, any common letter shared by two park types indicates that the two park types were found to belong to the same group.

### G. Park classification standard

This park classification standard is directly quoted from the NYC Department of Parks and Recreation, which is available at this link.

Table S-7 Park classification standard by the NYC Department of Parks and Recreation (DPR)

| Park Type | Description |
| --- | --- |
| Community Park | Parks with a combination of active and passive recreational facilities or specialized amenities that service more than one community district. The largest of these parks have a large natural area component. Community Parks are typically between 5 and 250 acres. |
| Flagship Park | These parks have a variety of active recreational facilities and include a large natural or landscaped component. Flagship Parks are some of the largest parks in the City and attract users from throughout the metropolitan region. They include, but are not limited to those joint interest areas that traverse multiple community districts. |
| Jointly Operated Playground | Playgrounds adjacent to public schools jointly operated by NYC DPR and the Department of Education |
| Nature Area | Vacant/unimproved area, including islands, which are not associated with other parks and contain natural features including forests, marshland, meadows, etc. |
| Neighborhood Park | Parks that are intended to serve the direct neighborhood in which they are located. Neighborhood Parks are typically up to 50 acres and may include passive/active recreational areas. |
| Playground | Standalone facilities under DPR jurisdiction and management consisting of playground equipment along with perhaps hard surface or turf sports areas. Typically under 5 acres and more than 50% of the total site. |
| Recreational Field/Courts | Sites consisting solely of hard surface/turf sports areas that are operated by DPR. |
| Triangle/Plaza | A landscaped or paved area usually in conjunction with the arterial or local street system. These sites are primarily developed for passive recreation use or to provide an open space amenity within the neighborhood. These sites are non-linear and may be developed to contain grass, trees, shrubs, cobblestone, fences, monuments, plaques, flagpoles, benches, game or picnic tables and drinking fountains, but they have no active recreational equipment. Smaller sites may be operated as Greenstreets, but are under the jurisdiction of DPR. These sites range in size, but are typically under 1 - acre. |

### H. Administrative orders regarding park/school closure and reopening in NYC

The timeline of these administrative orders was compiled from the government website and their social media account, where these orders were announced.


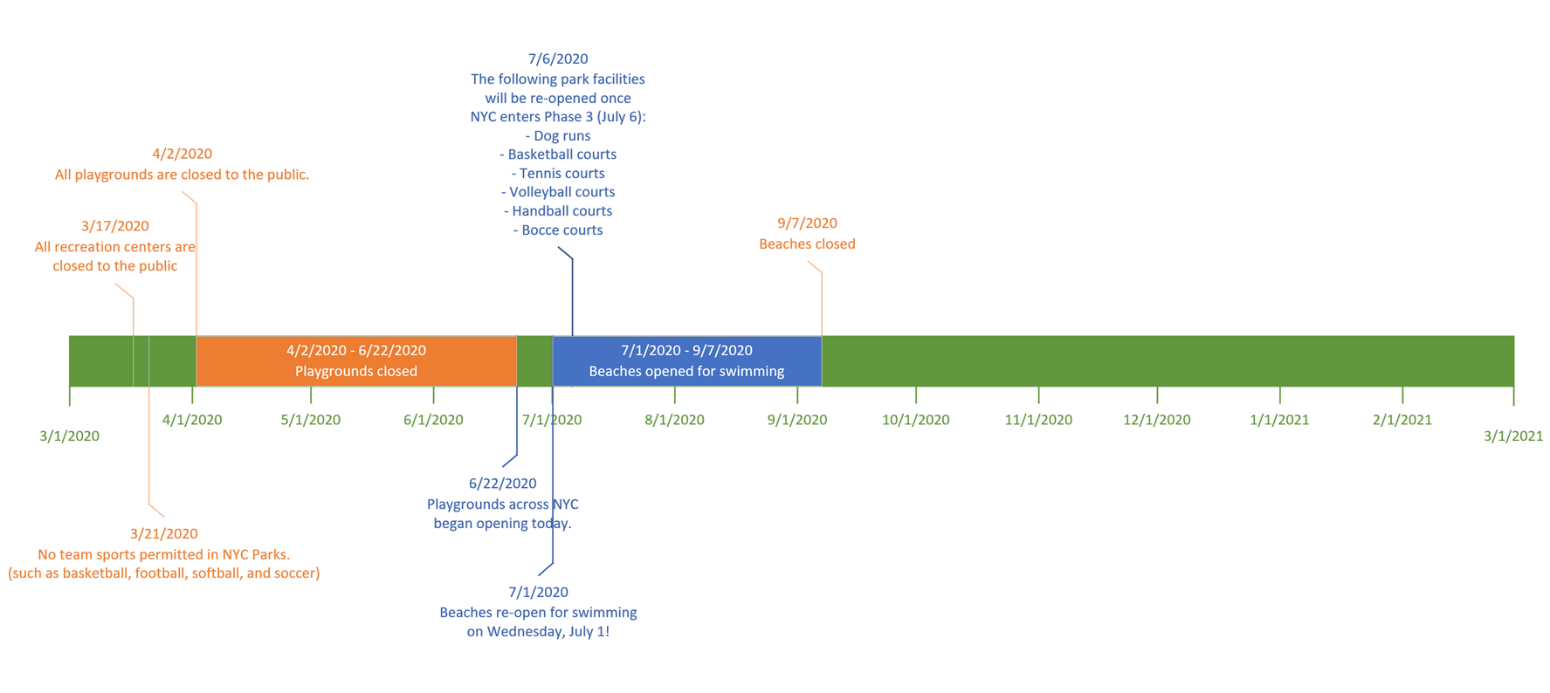


Figure S-3 Timeline of the closure and reopening of NYC parks


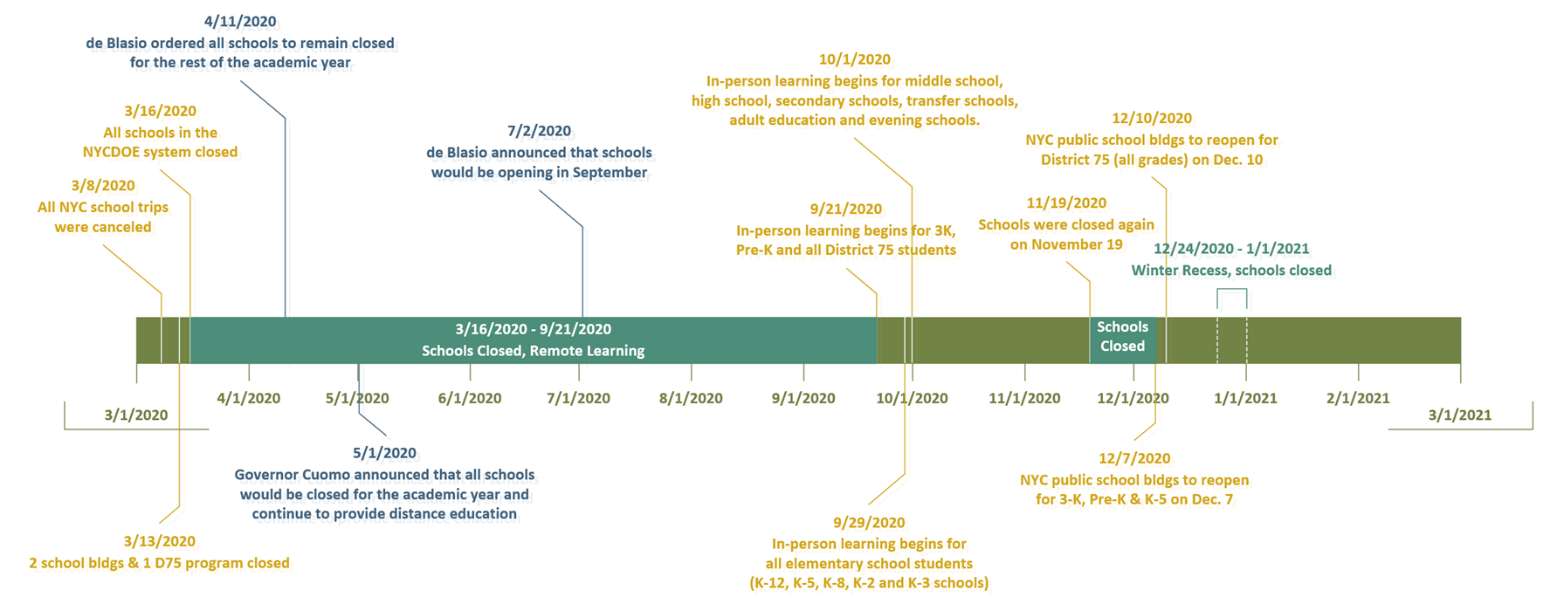


Figure S-4 Timeline of the closure and reopening of NYC schools

### I. Assessment about normalizing the visits for the change in SafeGraph’s device panel


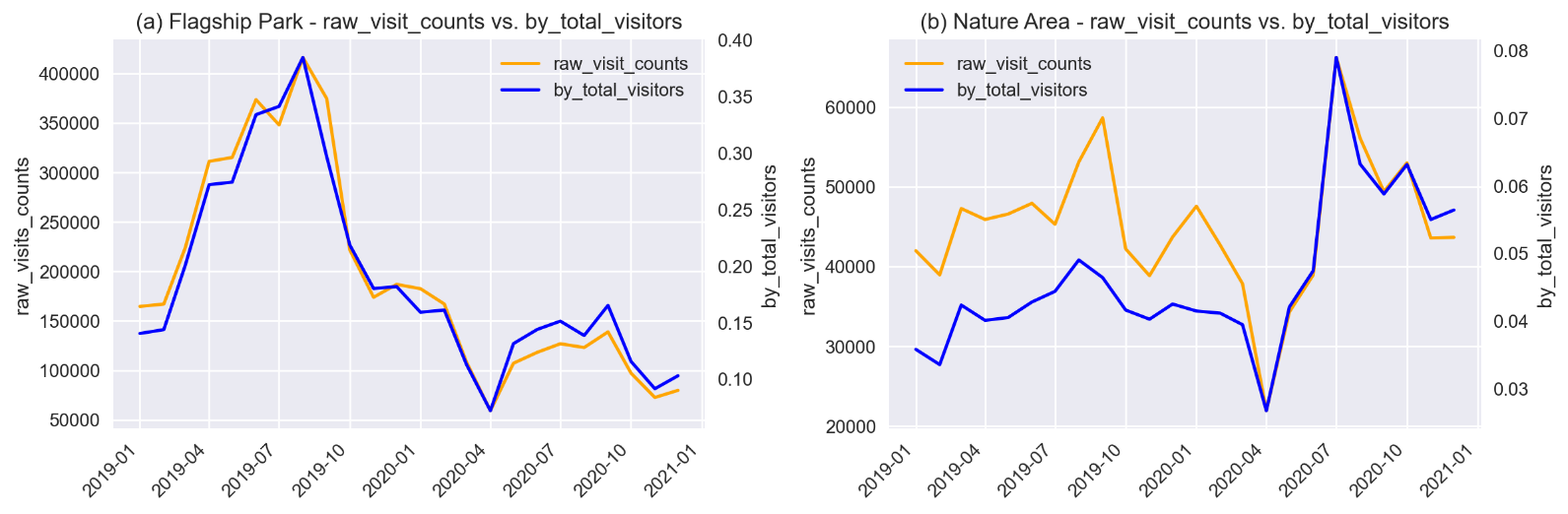


Figure S-5 Example figures of raw visits data vs. normalized visits data by total visitors. (a) Flagship Park (b) Nature Area

Table S-8 Pearson’s correlation coefficient between raw visits and normalized visits by total visitors for each park type

| Park Type | Community Park | Flagship Park | Jointly Operated Playground | Nature Area | Neighborhood Park | Playground | Recreation Field/Courts | Triangle/Plaza | Overall |
| --- | --- | --- | --- | --- | --- | --- | --- | --- | --- |
| Pearson’s R | 0.964 | 0.982 | 0.972 | 0.763 | 0.974 | 0.919 | 0.923 | 0.990 | 0.980 |
